# Mathematical problem solving is modulated by word priming

**DOI:** 10.1002/pchj.732

**Published:** 2024-02-01

**Authors:** Chuanlin Zhu, Zhao Zhang, Xiaoli Lyu, Yun Wang, Dianzhi Liu, Wenbo Luo

**Affiliations:** ^1^ School of Educational Science Yangzhou University Yangzhou China; ^2^ Institute of Psychology Weifang Medical University Weifang China; ^3^ Affiliated WuTaiShan Hospital of Medical College of Yangzhou University Yangzhou China; ^4^ School of Foreign Languages Suzhou University of Science and Technology Suzhou China; ^5^ School of Education Soochow University Suzhou China; ^6^ Research Center of Brain and Cognitive Neuroscience Liaoning Normal University Dalian China; ^7^ Key Laboratory of Brain and Cognitive Neuroscience Dalian China

**Keywords:** arithmetic performance, emotional word, estimation task, priming paradigm

## Abstract

This study aimed to explore the influence of word priming on mathematical problem solving. In two experiments, participants were required to finish multiplication estimation tasks with a specified estimation strategy under different word priming conditions (Experiment 1: concrete words vs. Experiment 2: abstract words). The results showed that: (1) under the concrete word priming condition, in comparison to neutral, positive word priming improved accuracies (ACCs) when using a down‐up strategy (e.g., doing 40 × 80 = 3200 for 43 × 78), while both positive and negative word priming reduced reaction time (RT); (2) under the abstract word priming condition, both positive and negative (vs. neutral) abstract word priming reduced RTs, while individuals’ ACCs of completing the estimation task were not influenced by valence. The present study showed that whether concrete words or abstract words were adopted as experimental stimuli, participants’ performance of completing mathematical problems was modulated by the valence of the priming word, which led us to develop a better understanding of how arithmetic performance is influenced by word processing.

## INTRODUCTION

Estimation refers to individuals producing approximate answers by using certain principles when solving a calculation problem (Hinault & Lemaire, 2017). Commonly used multiplication computational estimation strategies include rounding up (RU; i.e., do 40 × 70 for 31 × 67), rounding down (RD; i.e., do 30 × 60 for 31 × 67), and mixed round (MR), which includes down‐up (DU; i.e., do 30 × 70 for 31 × 67), and up‐down (UD; i.e., do 40 × 60 for 31 × 67) (Ai et al., 2017; Hinault & Lemaire, 2017; Liu et al., 2021). Numerous studies have shown that RU is harder than RD (Hinault et al., 2014; Taillan et al., 2015), and one review noted that the RD strategy was easiest, the MR (DU and UD) was of medium difficulty, and the RU was hardest (Hinault & Lemaire, 2016). However, few studies have compared the difficulty of using DU and UD strategies. One of the goals of the present study was to address this issue.

Researchers have found that individuals' estimation performance can be affected by factors such as central execution load (Yang et al., 2018), age (Lallement & Lemaire, 2021), cultural differences (Zhao et al., 2014), cognitive style (Zhang, Wang, et al., 2015), emotions (Fabre & Lemaire, 2019; Lallement & Lemaire, 2021; Liu et al., 2021), and so forth. A study by Liu et al. (2021) investigated the effects of explicit emotional priming on individuals’ arithmetic performance. Liu et al. (2021) asked participants to complete the two‐digit multiplication estimation task (TDME task) under different emotional priming conditions (happy, neutral, anger, and fear). According to the results, participants responded more quickly under the happy (vs. angry and fear) priming condition, and more quickly under the neutral priming condition than under the fear priming condition. Meanwhile, a study conducted by Zhu, Jiang, Li et al (2021) showed that both implicit fear and happy (vs. neutral and angry) priming enhanced estimation speed. In addition, Liu et al. (2021) and Zhu, Jiang, Li et al. (2021) found that the estimation ACC does not appear to be affected by emotional priming. Furthermore, negative emotional (vs. neutral) priming slowed down individuals’ estimation speed in another study (Lallement & Lemaire, 2021). Based on these studies, it appears that the valence of emotional priming influences estimation performance.

In previous studies, when investigating the influence of emotional experience on individuals' estimation performance, researchers mainly adopted emotional pictures (not face‐related pictures, but scenes of animals, landscapes, and life events) (Lallement & Lemaire, 2021) and facial expressions (Liu et al., 2021; Zhu, Jiang, Li, et al., 2021) as experimental material to induce participants’ emotional experience. However, in addition to emotional pictures and facial expressions, words are also an important medium for conveying emotions, and the processing processes of scene pictures, facial expressions, and words show certain similarities (Luo et al., 2010; Schacht & Sommer, 2009; Yi et al., 2015; Zhang et al., 2014). For example, in a previous study (Schacht & Sommer, 2009), words, pseudowords, and facial expression images were adopted as experimental stimuli, with both words and facial expression images classified into three types according to their valence, that is, negative, neutral, and positive. Participants were required to complete the lexical decision task (judge whether or not a given letter string was a correct word) and facial expression decision task (judge whether or not a given facial expression picture was intact), and the results showed that positive (vs. neutral and negative) words and facial expression images elicited posterior event‐related potential negativities in scalp distribution. Additionally, other studies showed that whether using scene pictures (Zhu et al., 2015), facial expression images (Luo et al., 2010), or words (Yi et al., 2015) as experimental material, individuals achieved a higher accuracy in identifying negative (vs. neutral and positive) stimuli. Because completing the scene picture and facial expression processing tasks affects individuals’ estimation performance, does the completion of the word processing task also affect individuals’ estimation performance?

Previous studies have shown that word priming can affect the completion of various cognitive tasks, such as word processing (Wu et al., 2021), affective picture processing (Wu et al., 2020), facial expression processing (Fan et al., 2015), memory processing (Xiao et al., 2012; Zhang, Liu, et al., 2015), and so on. For example, Yao and Wang (2013) examined the impact of the concreteness of words on affective priming. In their study, the target emotional (positive or negative) word was preceded by an emotional abstract/concrete word in a single trial. Participants were instructed to judge whether the target word was a pseudoword. They found that participants responded more slowly to negative (vs. positive) pairs in abstract–positive priming conditions. However, under concrete‐positive, concrete‐negative, and abstract‐negative priming conditions, participants' response speed to congruent pairs and incongruent pairs showed no significant difference. Other researchers found similar phenomena (Yao et al., 2016). These studies collectively indicate that, aside from valence, words' concreteness influences the affective priming effect.

Previous studies have shown that mathematical and semantic problem solving share some common brain networks, and the semantic network supports mathematical problem solving (Li, Tan, et al., 2019; Liu et al., 2019; Zhou et al., 2018). For example, mathematical problem solving results in brain activity in the left angular gyrus (AG), left middle temporal gyrus (MTG), and left inferior frontal cortex (IFG), while the AG, MTG and orbital part of the IFG are key regions of the semantic system (J. Liu et al., 2019). Given that the semantic network supports mathematical problem solving, it is reasonable to believe that the completion of the word processing task may affect an individual's mathematical performance. If so, would a participant's mathematical performance be affected by the priming words' valence and concreteness? These issues are still up in the air.

The present study aimed to investigate the influence of word priming on individuals’ mathematical performance. In Experiment 1, a within‐subjects design of 3 (concrete emotion word: positive, negative, and neutral) × 2 (estimation strategy: DU and UD) was adopted. The DU strategy denotes the down‐up strategy (e.g., do 40 × 80 for 43 × 78), while the UD strategy denotes the up‐down strategy (e.g., do 50 × 70 for 47 × 73). Participants were asked to finish the TDME tasks by using the UD or DU strategy under different concrete words priming conditions. The experimental design in Experiment 2 was similar to that in Experiment 1, except that abstract words were substituted for concrete ones. We decided to use two detached experiments based on the following considerations. First, we intended to investigate whether the influences of concrete and abstract words' valence on arithmetic performance were different, and thus concrete and abstract words were adopted in two detached experiments. Second, the pretest showed that the duration of a single experiment seemed too long, and the participants who completed the pretest reported they felt tired. Therefore, two detached experiments may contribute to alleviating the influence of fatigue. The present study included three sub‐goals. (1) Sub‐goal one: To investigate the difficulty of using the DU and UD strategies. According to working memory theory, both the DU and the UD strategy only require incrementing and maintaining one operand in working memory, and the DU and UD strategies consume the same amount of working memory resources (Uittenhove & Lemaire, 2012); thus, we hypothesized that no significant differences would be seen in the difficulties of using the DU and UD strategies. To be more specific, the corresponding ACCs and RTs of using the DU and the UD strategy to complete the estimation tasks would show no significant differences in both experiments (Hypothesis 1). (2) Sub‐goal two: To study the influence of the valence of the priming words on participants’ arithmetic performance. As mentioned above, the processing processes of scene pictures, facial expressions, and words show certain similarities (Luo et al., 2010; Schacht & Sommer, 2009; Yi et al., 2015), and the influences of scene picture and facial expression priming on arithmetic performance are modulated by valence (Fabre et al., 2022; Lemaire, 2022; Liu et al., 2021; Zhu, Jiang, Li, et al., 2021); we thus expected to find that the influence of word priming on arithmetic performance was modulated by valence (Hypothesis 2). (3) Sub‐goal three: To explore whether Hypothesis 2 holds only when concrete (but not abstract) words are adopted as priming stimuli. No precise predictions were made a priori for this sub‐goal, because it was meant to be exploratory.

## EXPERIMENT 1: THE PRIMING EFFECT OF POSITIVE AND NEGATIVE CONCRETE WORDS ON ARITHMETIC PERFORMANCE

### Participants

In a previous similar study, a two‐way interaction was found with a 3 (emotion type: positive, neutral, and negative) × 2 (estimation problem type: easy and hard) design (effect size η^2^_p_ = 0.26) (Fabre & Lemaire, 2019). Reference to the Fabre & Lemaire, (2019) a priori power analysis with MorePower 6.0.4 (Campbell & Thompson, 2012) indicated that a power of η^2^_p_ = 0.26 (α = 0.05, power level = 0.95) would need 24 participants, when focusing on the interaction between estimation strategy type (UD and DU) and emotion type (fear, neutral, and happy). In order to ensure a sufficiently large sample size, 42 (26 females) participants were randomly recruited from Yangzhou University. Their average age was 19.93 ± 1.34 (*M* ± *SD*) years old (range 18–24). All participants had normal/corrected‐to‐normal visual acuity. Prior to completing the experiment, participants were instructed to sign an informed consent form, which was consistent with the Declaration of Helsinki (1991). After finishing the experimental task, each of them received a small amount of money for participating. This study was approved by the Research Ethics Committee of the School of Educational Science of Yangzhou University (JKY‐2021030508).

### Materials

#### Words

Twenty‐seven two‐character words (e.g., “珠宝,” which means “jewel”) were selected from the CAWS (Chinese Affective Words System; Wang et al., 2008). These words were classified into three types according to their valence, that is, positive (e.g. “礼物,” which means “gift”), negative (e.g. “小偷,” which means “burglar”), and neutral (e.g. “房屋,” which means “house”), with each type including nine words. In the CAWS, for each word, participants had assessed the valence, arousal, familiarity, and concreteness using a 9‐point scale. On the 9‐point scale, “1” stands for “very unpleasant, calming, unfamiliar and abstract”, while “9” stands for “very pleasant, arousing, familiar and concrete”. The descriptive statistics (means and standard deviations) for the valence, arousal, familiarity, concreteness, and strokes are summarized in Table 1. A one‐way analysis of variance (ANOVA) of the five variables was conducted. The results indicated that the main effect of valence was significant, *F*(2, 24) = 96.918, *p* < .001; valence ratings significantly differed across all three types of words (*p*s < .001). The main effect of arousal was significant, *F*(2, 24) = 17.188, *p* < .001; the arousal levels of positive and negative words were higher (*p*s < .001) than those of neutral words, while the arousal levels of positive and negative words showed no significant difference (*p* > .05). The main effect of familiarity was not significant, *F*(2, 24) = 2.561, *p* = .098. The main effect of concreteness was not significant, *F*(2, 24) = 0.987, *p* = .387. The main effect of strokes (stroke means one of the lines of a letter of the word) was not significant, *F*(2, 24) = 1.955, *p* = .164.

**TABLE 1:** Statistical analysis of the selected words in Experiment 1 (M ± SD).

	Valence	Arousal	Familiarity	Concreteness	Strokes
Positive	6.87 ± 0.44	5.74 ± 0.35	5.85 ± 0.72	1.9 ± 0.39	16.33 ± 2.29
Negative	3.15 ± 0.65	5.81 ± 0.46	5.18 ± 0.67	2.77 ± 1.36	13.56 ± 3.43
Neutral	5.57 ± 0.62	4.15 ± 1.03	5.72 ± 0.61	2.26 ± 1.8	16.33 ± 4.3

In order to avoid the ease or difficulty of categorizing the priming words, and to attribute differences in dependent variables solely to valence, we conducted a separate procedure to check whether the three types of words were similar in ease or difficulty for participants. For this purpose, 10 trained participants (those who did not take part in the formal experiment), blind to the research purposes, were invited to complete the word rating task: they were required to judge the valence of the word and asked to press key “1” when the word was negative, to press key “2” when it was neutral, and to press key “3” when it was positive. We averaged the rating data across all participants for each word, and the ACC and RT of rating each type of word were measured by a one‐way ANOVA for the ACC and RT. The independent factor involved in the analysis was emotional valence (negative, neutral, positive). The results on ACC showed that the main effect of valence was not significant, *F*(2, 27) = 2.441, *p* = .106. The results on RTs showed that the main effect of valence was not significant, *F*(2, 27) = 0.177, *p* = .839. The results on ACC and RT together showed that there was no difference in the difficulty of recognizing the three types of concrete words.

#### TDME problems

One hundred and eight TDME problems (e.g., 32 × 67) were used in the present study, with 54 of them suitable for the UD strategy and the other 54 suitable for the DU strategy. The principles for choosing the TDME problems were consistent with previous studies (Zhu et al., 2023; Zhu, Jiang, Wang, et al., 2021; Zhu, Li, et al., 2022). To be more specific: (1) no operand had its closest decade equal to 10 (e.g., 13 × 47), or 100 (e.g., 24 × 98); (2) no operands had 0 (e.g., 41 × 70) or 5 (e.g., 35 × 58) as unit digits; (3) operands were not repeated in the decade (e.g., 42 × 49) or unit (e.g., 47 × 57); (4) no digits were repeated within operands (e.g., 44 × 67); (5) no tie problems were used (e.g., 36 × 36); and (6) the first operand was smaller than the second (e.g., 32 × 57), owing to Chinese preferred operand‐order‐specific representation; that is, they performed better while the first operand was smaller (vs. larger) than the second.

### Procedure

**FIGURE 1:**
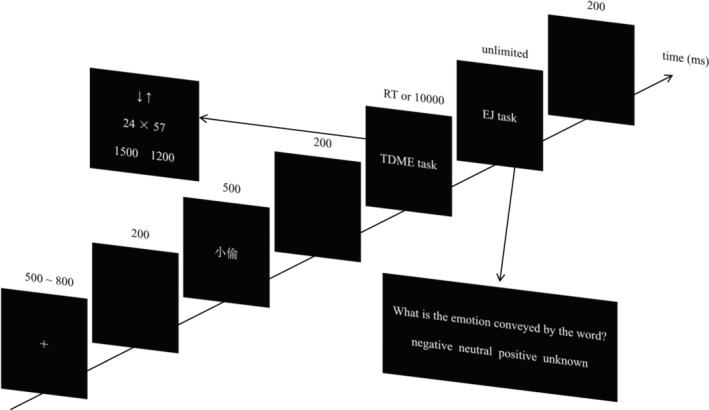
Illustration of one experimental trial in Experiment 1.

The experimental procedure of this study was programmed and presented with E‐prime 2.0 (Psychology Software Tools, Pittsburgh, PA, USA). The formal experiment consisted of four blocks, and each block contained 54 trials, for a total of 216 trials. As shown in Figure 1, in a single trial, the stimuli were presented in the following order: a white “+” (500 ~ 800 ms), a blank (200 ms), a word (500 ms), a blank (200 ms), the TDME task (RT or 10000 ms), next, the emotion judgment task (EJ task, unlimited), then a blank (200 ms). In the TDME task, the TDME question and two alternative answers were presented simultaneously. Both UD and DU strategies were used to calculate the alternative answers. Participants were told that only the DU and UD strategies could be adopted, and they were told to find out the correct answer with the specified strategy. “↑↓” or “↓↑” was presented above the TDME question as a cue, and the cue was matched with the TDME question type (e.g., “↓↑” was presented together with 24 × 57). Before completing the experimental task, all participants were told to use the estimation strategy suggested by the arrow cue to complete the TDME task, with “↑↓” representing the UD strategy and “↓↑” representing DU strategy. Participants were told to press the “F” (“J”) key with their left (right) index finger, if the correct answer was presented on the left (right) side. In the EJ task, participants were told to judge the valence of the word. From left to right on the screen, there were four optional answers: “negative,” “neutral,” “positive,” and “unknown.” The corresponding keys were as follows: press “D” with the left middle finger; press “F” with the left index finger; press “J” with the right index finger; and press “K” with the right middle finger. In both tasks, the probability of the correct answer appearing at the corresponding location was equal, and the correct answer was not presented in the same position continuously more than 3 times.

The present study was conducted in a sound‐attenuated room. Subjects sat about 90 cm in front of the monitor, which had a resolution of 1024 × 768 pixels and a refresh rate of 100 Hz. Six experimental conditions were included in Experiment 1, because a 3 (concrete emotion word: positive, negative, and neutral) × 2 (estimation strategy: UD and DU) experimental design was adopted. A pseudo‐random experimental design was adopted in this study; each experimental condition would not appear sequentially more than 3 times. Before the start of the formal experiment, participants were asked to place the left middle finger, left index finger, right index finger, and right middle finger on the four keys “D,” “F,” “J,” and “K” in sequence, and they were told to complete the experimental tasks as accurately and quickly as possible. To ensure that the experiment was fully understood by the participants, 12 practice trials (2 trials for each condition) were carried out before the formal experiment. Feedback was given on participants’ responses in the practice phase (but not in the formal experiment phase). Only when the participants had completed the two tasks with a correct rate of not less than 80% in the practice phase did the formal experiment start. The words and TDME questions used in the practice phase were not used in the formal experiment. To reduce the effect of fatigue, participants rested for 2 min after finishing each block, and then performed the next one.

### Data analysis

The TDME task‐related results were measured by 3 × 2 repeated measure ANOVAs. The independent variables were emotion types (positive, negative, and neutral) and estimation strategy types (UD and DU); the dependent variables were the accuracy and reaction time for completing the TDME task. The EJ task‐related results were measured by one‐way ANOVAs. The independent variables were emotion types (positive, negative, and neutral); the dependent variables were the accuracy and reaction time for completing the EJ task. Statistical analysis was conducted using SPSS 16.0 (SPSS Inc., Chicago, IL, USA). The Greenhouse–Geisser correction was applied to the *p*‐values when the number of degree of freedom did not meet the spherical test hypothesis. Additionally, all post‐hoc tests were conducted using the Bonferroni method. Trials were accepted for analysis only when participants gave correct response to the EJ tasks. Trials with RT < 3SD below the mean RT or with RT > 3SD above the mean RT for an individual participant for each condition were also excluded. In Experiment 1, the excluded trials per condition were as follows: 6.09 trials under the positive priming condition while using the DU strategy (positive‐DU), 2.69 trials under the negative‐DU condition, 7.81 trials under the neutral‐DU condition, 6.21 trials under the positive‐UD condition, 3.10 trials under the negative‐UD condition, and 7.91 trials under the neutral‐UD condition. Partial eta‐squared (η_P_^2^) was adopted to describe effect sizes (Cohen, 1988). Based on null hypothesis significance testing (NHST), we adopted the *p*‐values to make inferences. As is known, *p* > 0.05 means the absence of evidence for the specified effect, rather than the absence of the specified effect; we adopted the XLSTAT equivalence test (conducted using XLSTAT 2021.2.2 [Data Analysis and Statistical Solution for Microsoft Excel, Addinsoft, Paris, France]) to provide evidence in support of the null results, because the XLSTAT equivalence test (TOST, two‐one‐sided‐test) allows us to test if two products are equivalent. For the sake of brevity, we report the equivalence test results in Data S1.

### Results

#### The TDME task

**FIGURE 2:**
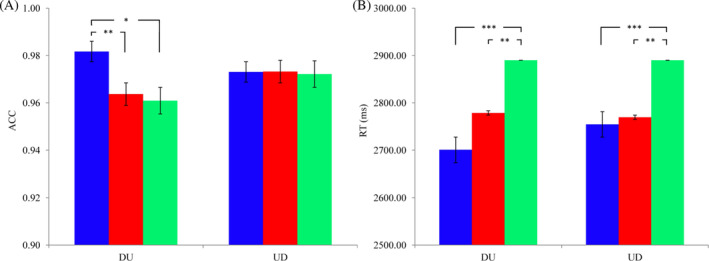
Accuracy (ACC) (A) and reaction time (RT) (B) of completing the two‐digit multiplication estimation (TDME) tasks. “*” means “*p* < .05”, “**” means “*p* < .01”, “***” means “*p* < .001”. Different colored bars represent different word priming conditions: blue = positive, red = negative, and green = neutral.

ACC: The ACC results showed a ceiling effect (ACCs > 0.96). The 3 (emotion types: positive, negative, and neutral) × 2 (estimation strategies: DU and UD) repeated‐measure ANOVAs revealed a significant interaction between emotion type and estimation strategy, *F*(2, 82) = 4.390, *p* = .015, η_P_^2^ = 0.097. The main effect of emotion type was not significant, *F*(2, 82) = 2.258, *p* = .126, η_P_^2^ = 0.052. The main effect of estimation strategy was not significant, *F*(1, 41) = 1.156, *p* = .289, η_P_^2^ = 0.027. Simple effect analysis showed that when using the DU strategy, ACC under the positive priming condition (*M* ± *SD*, 0.982 ± 0.054) was higher than those under neutral (0.961 ± 0.103, *p* = .031) and negative (0.964 ± 0.081, *p* = .008) conditions, and there was no significant difference between neutral and negative conditions (*p* = .658). Meanwhile, for the UD strategy, ACC under different priming conditions showed no significant difference (*p*s > .05). See Figure 2A.

RT: The 3 (emotion types: positive, negative, and neutral) × 2 (estimation strategies: DU and UD) repeated‐measure ANOVAs revealed a significant main effect of emotion type, *F*(2, 82) = 14.345, *p* < .001, η^2^_p_ = 0.259. The main effect of estimation strategy was not significant, *F*(1, 41) = 0.192, *p* = .664, η_P_^2^ = 0.005. The interaction between emotion type and estimation strategy was not significant, *F*(2, 82) = 1.204, *p* = .305, η_P_^2^ = 0.029. Post‐hoc pair‐wise comparisons showed that, compared with the neutral priming condition (*M* ± *SD*, 2890.035 ± 126.261), individuals’ RTs under positive (2727.639 ± 112.847, *p* < .001) and negative (2774.114 ± 117.267, *p* = .002) priming conditions were shorter, but the latter two conditions showed no significant difference (*p* = .397). See Figure 2B.

Experiment 1 indicated that both ACC (both for DU and UD) and RT (mainly for UD) in completing the TDME tasks were influenced by the valence of the emotional priming word, and the ACC results showed a ceiling effect. A previous study exploring the influence of emotional picture processing on individuals’ arithmetic performance showed that negative (positive) priming sped up (slowed down) an individual's response speed (Fabre & Lemaire, 2019), which partly supported our results. Experiment 1 suggested that arithmetic performance was modulated by the valence of a concrete word. In Experiment 2, we tested whether such effects of concrete word prime effects would be observed for abstract word primes.

#### The EJ task

ACC: A one‐sample *t*‐test showed that individuals’ ACCs in completing the EJ task under all conditions were significantly higher (*ps <* .001) than the probability level (0.25), which indicated that the participants successfully completed the EJ task. The results of a one‐way ANOVA revealed a significant main effect of emotion type, *F*(2, 82) = 13.188, *p <* .001, η_P_^2^ = 0.243. The ACCs under the negative priming condition were higher (*ps <* .001) than those under the positive and neutral priming conditions, while the latter two showed no significant difference (*p* = .571). The ACCs of completing the EJ task under different conditions are shown in Table 2.

**TABLE 2:** ACC and RT of completing the EJ task in Experiment 1 (*M* ± *SD*).

	Positive	Negative	Neutral
ACC	0.83 ± 0.15	0.92 ± 0.08	0.78 ± 0.13
RT	682.36 ± 313.65	652.10 ± 296.90	691.78 ± 307.90

Abbreviations: ACC = accuracy; EJ = emotion judgment; RT = reaction time.

RT: The results of a one‐way ANOVA showed that the main effect of emotion type was not significant, *F*(2, 82) = 2.256, *p* = .111, η^2^_p_ = 0.052. The RTs for completing the EJ task under different conditions are shown in Table 2.

## EXPERIMENT 2: THE PRIMING EFFECT OF POSITIVE AND NEGATIVE ABSTRACT WORDS ON ARITHMETIC PERFORMANCE

### Participants

The method of deciding the sample size was the same as that in Experiment 1. Forty‐two (25 females) students were randomly recruited from Yangzhou University, with an average age of 19.98 ± 0.84 (*M* ± *SD*) years old (range 18–22). All participants had normal/corrected‐to‐normal visual acuity, and did not suffer from brain trauma or mental illness. All participants were told to sign an informed consent form before completing the experimental task, which was consistent with the Declaration of Helsinki (1991). After finishing the experimental task, all of them received a small amount of money for their participation. The study was approved by the Research Ethics Committee of the School of Educational Science of Yangzhou University (JKY‐2021030508).

### Materials

#### Words

As in Experiment 1, 27 two‐character abstract words (e.g., “创新,” which means “innovate”) were selected from the CAWS (Wang et al., 2008). These words were classified into three types according to their valence, that is, positive (e.g., “信念,” which means “faith”), negative (e.g., “羞耻,” which means “shameful”), and neutral (e.g., “规范,” which means “criterion”), with each type including nine words. The descriptive statistics (means and standard deviations) for the valence, arousal, familiarity, concreteness, and strokes are summarized in Table 3. A one‐way ANOVA of the five variables was conducted. The results showed that the main effect of valence was significant, *F*(2, 24) = 145.566, *p* < .001, and valence ratings significantly differed across all three types of words (*p*s < .001). The main effect of arousal was significant, *F*(2, 24) = 12.140, *p* < .001. The arousal levels of positive (*p* < .001) and negative (*p* = .004) words were higher than those of neutral words, while there was no significant difference between positive and negative words (*p* > .05). The main effect of familiarity was not significant, *F*(2, 24) = 2.558, *p* = .098. The main effect of concreteness was not significant, *F*(2, 24) = 0.267, *p* = .768. The main effect of strokes was not significant, *F*(2, 24) = 0.242, *p* = .787.

**TABLE 3:** Statistical analysis of the selected words in Experiment 2 (*M* ± *SD*).

	Valence	Arousal	Familiarity	Concreteness	Strokes
Positive	6.83 ± 0.17	5.69 ± 0.42	5.67 ± 0.43	4.66 ± 0.28	17.22 ± 4.15
Negative	3.09 ± 0.41	5.42 ± 0.37	5.07 ± 0.90	4.87 ± 0.65	16.67 ± 3.12
Neutral	5.02 ± 0.67	4.54 ± 0.70	5.55 ± 0.26	4.73 ± 0.81	17.89 ± 3.86

As in Experiment 1, 10 trained participants (who did not take part in the formal experiment) were invited to complete the word rating task, with the rating procedure the same as that in Experiment 1. The ACCs and RTs of rating each type of word were measured by a one‐way ANOVA. The independent factor involved in the analysis was emotional valence (negative, neutral, positive). The results for ACC showed that the main effect of valence was not significant, *F*(2, 27) = 0.794, *p* = .462. The results for RT showed that the main effect of valence was not significant, *F*(2, 27) = 0.759, *p* = .478. The results for ACC and RT together showed that there was no difference in the difficulty of recognizing the three types of abstract words.

#### TDME problems

The TDME problems used in Experiment 2 were the same as those used in Experiment 1.

### Procedure

The procedure of Experiment 2 was the same as that of Experiment 1. The detailed process is shown in Figure 3.

**FIGURE 3:**
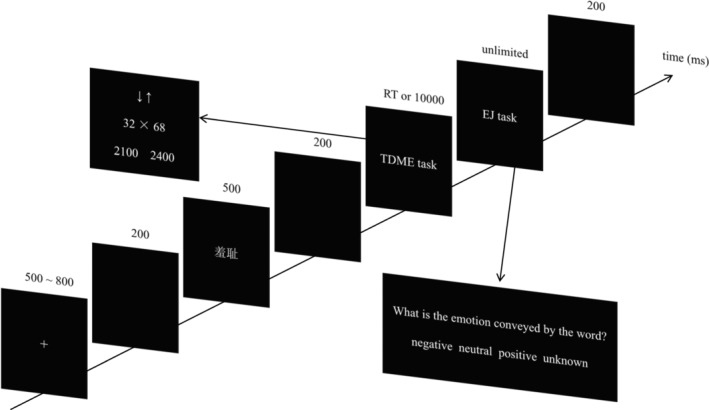
Illustration of one experimental trial in Experiment 2.

### Data analysis

The data analysis method was the same as that in Experiment 1. In Experiment 2, the excluded trials under each condition were as follows: 4.88 trials under the positive priming condition while using the DU strategy (positive‐DU), 6.36 trials under the negative‐DU condition, 8.02 trials under the neutral‐DU condition, 5.26 trials under the positive‐UD condition, 6.33 trials under the negative‐UD condition, and 8.10 trials under the neutral‐UD condition.

### Results

#### The TDME task

ACC: The ACC results showed a ceiling effect (ACCs > 0.97). The 3 (emotion types: positive, negative, and neutral) × 2 (estimation strategies: DU and UD) repeated‐measure ANOVAs showed that the main effect of emotion type was not significant, *F*(2, 82) = 0.238, *p* = .789, η^2^_p_ = 0.006. The main effect of estimation strategy was not significant, *F*(1, 41) = 0.430, *p* = .516, η^2^_p_ = 0.010. The interaction between emotion type and estimation strategy was not significant, *F*(2, 82) = 0.229, *p* = .796, η^2^_p_ = 0.006. See Figure 4A.

**FIGURE 4:**
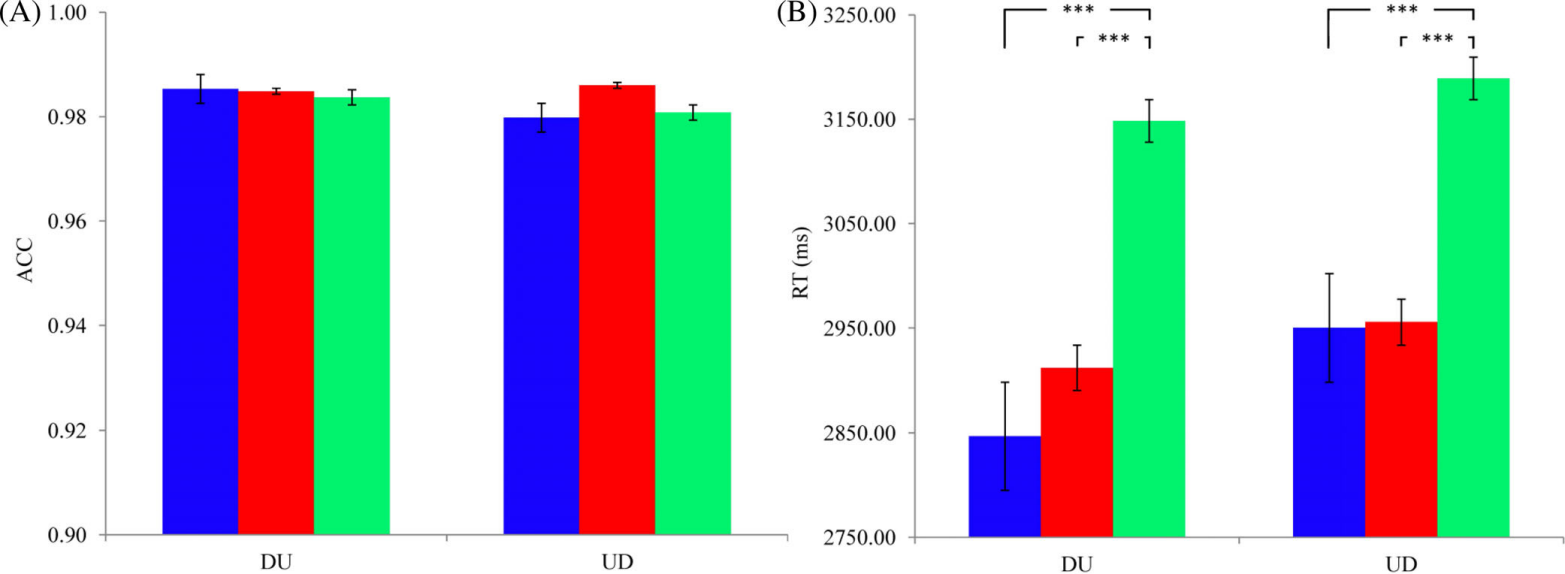
Accuracy (ACC) (A) and reaction time (RT) (B) of completing the two‐digit multiplication estimation (TDME) tasks. “***” means “*p* < .001”. Different colored bars represent different word priming conditions: blue = positive, red = negative, and green = neutral.

RT: The 3 (emotion types: positive, negative, and neutral) × 2 (estimation strategies: DU and UD) repeated‐measure ANOVAs revealed a significant main effect of emotion type, *F*(2, 82) = 18.202, *p* < .001, η^2^_p_ = 0.307. The main effect of estimation strategy was not significant, *F*(1, 41) = 3.511, *p* = .068, η^2^_p_ = 0.079. The interaction between emotion type and estimation strategy was not significant, *F*(2, 82) = 0.489, *p* = .615, η^2^_p_ = 0.012. Post‐hoc pair‐wise comparisons showed that, compared with the neutral priming condition (*M* ± *SD*, 3168.816 ± 127.618), individuals’ RTs under positive (2898.525 ± 95.939, *p* < .001) and negative (2934.062 ± 98.723, *p* < .001) priming conditions were shorter, but the latter two conditions showed no significant difference (*p* = .764). See Figure 4B.

#### The EJ task

ACC: A one‐sample *t*‐test showed that individuals’ ACCs of completing the EJ task under all conditions were significantly higher (*ps <* .001) than probability level (0.25), which suggests that the participants successfully completed the EJ task. The results of a one‐way ANOVA revealed that the main effect of emotion type was not significant, *F*(2, 82) = 2.998, *p* = .070, η^2^_p_ = 0.068. The ACCs of completing the EJ task under different conditions are shown in Table 4.

**TABLE 4:** ACC and RT of completing the EJ task in Experiment 2 (*M* ± *SD*).

	Positive	Negative	Neutral
ACC	0.86 ± 0.14	0.82 ± 0.14	0.78 ± 0.21
RT	683.41 ± 287.19	705.65 ± 359.14	792.57 ± 426.02

Abbreviations: ACC = accuracy; EJ = emotion judgment; RT = reaction time.

RT: The results of a one‐way ANOVA revealed a significant main effect of emotion type, *F*(2, 82) = 4.737, *p =* .017, η^2^_p_ = 0.104. The RTs under the negative priming condition were shorter (*p =* .017) than under the neutral priming condition, and the RTs under the positive priming conditions and those under the negative and neutral ones showed no significant difference (*ps* > .05). The RTs of completing the EJ task under different conditions are shown in Table 4.

## GENERAL DISCUSSION

As far as we know, the present study is the first to explore the influence of emotional word priming on arithmetic performance. Participants were asked to complete TDME tasks with a DU or UD strategy, under concrete (Experiment 1) or abstract (Experiment 2) word (positive, neutral, or negative two‐character word) priming conditions. Combining the NHST with the XLSTAT equivalence test, ACCs and RTs for completing the TDME tasks were analyzed. The results showed that: (a) under concrete word priming conditions (Experiment 1), compared with neutral, both positive and negative priming contributed to shortening RTs (for DU and UD), but only positive priming contributed to improving ACC (for DU); (b) under abstract word priming conditions (Experiment 2), compared with neutral, both positive and negative priming contributed to shortening RTs. However, there were no differences between ACCs under different conditions. Additionally, in both experiments, when participants were asked to complete the TDME tasks by using the DU or UD strategy, the corresponding ACCs and RTs showed no significant differences, which indicates that the difficulties of using the DU and the UD strategy showed no significant differences. Hypothesis 1 was confirmed.

In Experiment 1, compared with neutral, individuals’ response speed was quicker under the negative condition, which was also found in previous studies (Fabre & Lemaire, 2019; Zhu, Jiang, Li, et al., 2021). Additionally, processing a positive stimulus contributed to improving both ACC and RT, which was partly found in previous studies (Lemaire & Brun, 2018; Zhu, Jiang, Li, et al., 2021). Previous studies (Liu et al., 2021; Zhu, Jiang, Li, et al., 2021) showed that ACC was not influenced by emotional facial expression priming, when participants were asked to use only one estimation strategy. However, the present study indicated that participants' ACC was modulated by the valence of the priming words when they were asked to use two different estimation strategies, which reflects the necessity of adopting multiple estimation strategies when investigating the influence of emotional priming on an individual's arithmetic performance. In Experiment 2, compared with neutral, processing both positive and negative stimuli contributed to improving individuals’ response speed, which was consistent with the results of Experiment 1 and was partly supported by previous studies (Fabre & Lemaire, 2019; Zhu, Jiang, Li, et al., 2021). Previous studies showed that participants’ arithmetic performances were affected by the valence of emotional priming stimuli, when using facial expression images (Liu et al., 2021; Zhu, Jiang, Li, et al., 2021; Zhu, Zhao, et al., 2022) and scene pictures (Fabre et al., 2022; Fabre & Lemaire, 2019; Lemaire, 2022) as emotional priming stimuli; in the present study, a similar phenomenon was found again, even though the emotional priming stimuli were replaced with words, regardless of their concreteness (concrete and abstract). Therefore, these findings together indicate that the modulation by valence of the influence of emotional words priming on arithmetic performance is a universal and stable phenomenon.Hypothesis 2 was confirmed.

Experiment 1 and Experiment 2 together showed that both positive and negative word priming contributed to improving the individual's arithmetic performance, which may be explained by the broaden‐and‐build theory (Fredrickson, 2001; Fredrickson & Branigan, 2005) and the negative bias hypothesis (Li, Zhu, et al., 2019; Luo et al., 2010; Zhang et al., 2014). The broaden‐and‐build theory postulates that positive emotional experience contributes to broadening temporary thought‐action repertoires, which in turn serve for building an individual's lasting personal resources, including physical resources, intellectual resources, psychological resources, and so on. In other words, a positive emotional experience may contribute to broadening one's attention scope, which may provide more attention resources for completing the TDME task, and thus contribute to improving cognitive performance (Vieillard & Bigand, 2014; Zhang et al., 2022). Additionally, the negative bias hypothesis posits that individuals process negative (vs. neutral) stimuli faster, with a lower cognitive resource cost (Li, Zhu, et al., 2019; Luo et al., 2010; Zhang et al., 2014), which contributes to providing more attention resources to complete the TDME tasks, so that the performance of completing the TDME tasks was better under the negative (vs. neutral) word priming condition. Furthermore, it might be argued that categorizing a word as neutral is probably much more resource‐demanding than classifying words as positive or negative, which may explain the positive/negative priming effect. However, the word rating task showed that there was no difference in recognizing the three types of concrete/abstract words, and thus it is reasonable to rule out the possible alternative explanation mentioned above.

Additionally, we were interested in participants' performance in completing the EJ task. The results showed that when processing concrete words, participants achieved higher ACCs for negative (vs. neutral and positive) words, but the corresponding RTs showed no significant difference (Experiment 1). However, when processing abstract words, participants identified negative (vs. neutral) words faster, while the corresponding ACCs showed no significant difference (Experiment 2). These results reflect the influence of valence on word processing; that is, participants performed better in identifying negative (vs. neutral) words, which is partly supported by previous studies (Yi et al., 2015; Zhang et al., 2014). Additionally, the cross‐experiment analysis results showed that participants' ACCs for processing the concrete (vs. abstract) words were higher, and this phenomenon occurred only when processing negative (but not neutral and positive) words, which may be due to word processing being influenced not only by valence (Vigliocco et al., 2014) but also by concreteness (Luo & Qi, 2022). Specifically, compared with concrete words, abstract words could evoke more emotional information, which may require more cognitive resources (Andrews et al., 2009), suggesting that when studying the effect of word priming on individuals’ performance of completing various cognitive tasks, the influence of valence and concreteness should be fully considered.

It should be noted that the results of this study should be interpreted with caution, considering that the results of the word rating tasks were partly consistent with those of previous studies. Researchers have not yet reached a consensus on individuals’ performance for completing word processing tasks. For example, in a previous study (Schindler & Kissler, 2016), participants were required to judge the valence of a given noun (positive, negative, or neutral): the results showed that participants’ ACC and RT for identifying the valence of different words (positive, negative, and neutral) showed no significant difference, which is supported by our word rating results. Meanwhile, in relation to a similar word processing task (judging the valence of the given word), another study (Yi et al., 2015) showed that the accuracy of recognizing negative words was higher than that for positive and neutral ones, while there was no significant difference between positive and neutral words. This inconsistency may be caused by the fact that neither of the two studies mentioned above nor our study distinguished between emotion‐label words and emotion‐laden words. Emotion‐label words refer to words that describe feelings or affective states (e.g., joy), while emotion‐laden words refer to words that relate to emotions without explicitly referring to affective states (e.g., tomb) (Sabater et al., 2022). Previous studies showed that participants’ performance on word processing tasks was affected by word type (emotion‐label words vs. emotion‐laden words) (Zhang et al., 2019). Adopting the flanker task, Zhang et al. (2019) investigated participants’ performance on processing emotion‐label words and emotion‐laden words: participants were asked to judge the valence of target words that were vertically surrounded by words with the same (congruent) or different (incongruent) valence as being negative or positive. The results showed that participants achieved a higher ACC when recognizing negative (but not positive) emotion‐laden words than when recognizing negative emotion‐label words, and they processed negative (but not positive) emotion‐laden words faster than emotion‐label words. Therefore, we speculate that the fact that the results of the word rating tasks in the present study seem to be partly inconsistent with those of previous studies may be because we did not distinguish between emotion‐label words and emotion‐laden words.

Previous studies mainly focused on the impact of facial expression and scene picture priming on individuals’ estimation performance, but the present study adopted word priming to extend the research scope to the level of emotional words. Compared with facial expressions and scene pictures, word priming provides a different way of stimulating emotions. The former (facial expressions and scene pictures) convey information with more direct biologically related cues, while words convey emotions on a symbolic level and more clearly convey emotional information (Schacht & Sommer, 2009; Zhang et al., 2014). Additionally, previous studies (Bai et al., 2005; Gong et al., 2011) showed that although some facial expression and emotional picture database (such as the NimStim set of facial expressions and IAPS) showed good reliability and validity, their effectiveness is still affected by culture. For example, when viewing a picture showing an old woman eating with the help of a middle‐aged woman, Chinese college students rated it more positively than did the National Institute of Mental Health (the developer of the IAPS) samples, which indicates the differences in understanding aging and self‐care in different cultures. However, word processing is less affected by cultural factors; for example, words' semantic representations seem similar in Chinese, English, and Spanish (Yao et al., 2017), and through emotional word priming we can more directly manipulate and measure participants’ emotional states. Therefore, the present study has contributed to deepening our knowledge of how individuals’ arithmetic performance is affected by emotional priming. Previous studies showed that the issue of whether age could moderate the link between emotional experience and arithmetic performance or not is a complex topic. For example, some studies showed that the influence of emotional experience on arithmetic performance was modulated by age (Lallement & Lemaire, 2021; Lemaire et al., 2019), while a recent study showed the opposite results (Lemaire, 2021). Considering that only young adults were tested in the present study, future studies could verify whether the present study's findings also apply to older adults. Additionally, previous studies (Luo et al., 2009; Muluh et al., 2011) showed that the potential neural mechanisms involved in addition, subtraction, multiplication, and division estimation tasks are not exactly the same. Therefore, it would be interesting to see whether the findings of this study hold true for other arithmetic problems (e.g., addition problems).

## CONCLUSION

The influence of word priming on individuals’ arithmetic performance was explored in this study. We found that under concrete word priming, positive (vs. neutral and negative) word priming improved ACC, and both positive and negative word priming contributed to shortening individuals’ RTs (Experiment 1). However, under abstract word priming, individuals’ ACCs of completing the TDME task were not influenced by valence, while both positive and negative words shortened RTs (but not ACCs, Experiment 2). These findings indicated that the modulation by valence of the influence of emotional words priming on arithmetic performance, regardless of the concreteness (concrete and abstract). This study and previous research together indicate that the influence of emotional priming on arithmetic performance is modulated by valence is a universal and stable phenomenon, when using different experimental stimuli (words, facial expression images, or scene pictures).

## CONFLICT OF INTEREST STATEMENT

The authors declare there are no conflicts of interest.

## ETHICS STATEMENT

This study was approved by the Research Ethics Committee of the School of Educational Science of Yangzhou University (JKY‐2021030508), and written informed consent was obtained from the participants.

## Supporting information


**Data S1.** Supplementary Information.

## Data Availability

The data that support the findings of this study are available on request from the corresponding author. The data are not publicly available owing to privacy or ethical restrictions.
