# Supplementary material for: Mathematical problem solving is modulated by word priming
Source: Psych J. 2024 Feb 1;13(3):465–76. doi: 10.1002/pchj.732 (PMC11169762; doi:10.1002/pchj.732)
Supplement: Supplementary file 1 — Data S1. Supplementary Information. [file PCHJ-13-465-s001.docx]

**Math problem solving is modulated by word priming**

Chuanlin Zhu^a*†^, Zhao Zhang^b†^, Xiaoli Lyu^c^, Yun Wang^d^,

Dianzhi Liu^e^, Wenbo Luo^f,g*^

1. School of Educational Science, Yangzhou University, Yangzhou, 225002, China
2. Institute of Psychology, Weifang Medical University, Weifang, 261000, China
3. Affiliated WuTaiShan Hospital of Medical College of Yangzhou University, Yangzhou, Jiangsu, 225003, China
4. School of Foreign Languages, Suzhou University of Science and Technology, Suzhou, 215009, China
5. School of Education, Soochow University, Suzhou 215123, China
6. Research Center of Brain and Cognitive Neuroscience, Liaoning Normal University, Dalian 116029, China
7. Key Laboratory of Brain and Cognitive Neuroscience, Liaoning Province, Dalian 116029, China

*: corresponding authors. †: Co-first authors.

Correspondence and requests for reprints to:

Dr. Chuanlin Zhu, School of Educational Science, Yangzhou University, Yangzhou, 225002; Tel: +86 411 87975536 (China (0514) 87975536), E-mail: [psyclzhu@yzu.edu.cn](mailto:psyclzhu@yzu.edu.cn)

Dr. Wenbo Luo, Research Center of Brain and Cognitive Neuroscience, Liaoning Normal University, Dalian, 116029, Tel: +86 411 8215 3336 (China (0411) 8215 3336), E-mail: [luowb@lnnu.edu.cn](mailto:luowb@lnnu.edu.cn)

**Supplemental Materials**

**1. The XLSTAT equivalence test results of experiment 1**

The equivalence tests results for ACC of completing the MCE task were shown in Table S1. The TOST (two-one-sided-test) interval was [-0.001, 0.001], the 95% confidence interval, and the total *p* value under different conditions were as follows.

Table S1 The 95% confidence interval on the difference between the means

|  | | Lower bound  (95%) | Upper bound  (95%) | The total *p*-value |
| --- | --- | --- | --- | --- |
| strategy | DU - UD | -0.036 | 0.028 | 0.621 |
| emotion | negative - positive | -0.038 | 0.020 | 0.751 |
|  | neutral - positive | -0.044 | 0.022 | 0.762 |
|  | neutral - negative | -0.039 | 0.035 | 0.562 |
| UD | negative - positive | -0.030 | 0.030 | 0.523 |
|  | neutral - positive | -0.033 | 0.031 | 0.547 |
|  | neutral - negative | -0.037 | 0.035 | 0.546 |

The equivalence tests results for RT of completing the MCE task were shown in Table S2. The TOST interval was [100, 100], the 95% confidence interval, and the total *p* value under different conditions were as follows.

Table S2 The 95% confidence interval on the difference between the means

|  | | Lower bound  (95%) | Upper bound  (95%) | The total *p* -value |
| --- | --- | --- | --- | --- |
| strategy | DU - UD | -348.899 | 319.294 | 0.752 |
| DU | negative - positive | -258.013 | 413.846 | 0.552 |
|  | neutral - positive | -157.727 | 536.331 | 0.695 |
|  | neutral - negative | -239.534 | 462.305 | 0.526 |
| UD | negative - positive | -307.093 | 337.159 | 0.699 |
|  | neutral - positive | -201.702 | 472.683 | 0.583 |
|  | neutral - negative | -224.316 | 465.231 | 0.547 |

**2. The XLSTAT equivalence test results of experiment 2**

The equivalence tests results for ACC of completing the MCE task were shown in Table S3. The TOST interval was [-0.001, 0.001], the 95% confidence interval, and the total *p* value under different conditions were as follows.

Table S3 The 95% confidence interval on the difference between the means

|  | | Lower bound  (95%) | Upper bound  (95%) | The total *p*-value |
| --- | --- | --- | --- | --- |
| strategy | DU - UD | -0.006 | 0.011 | 0.626 |
| emotion | negative - positive | -0.014 | 0.008 | 0.763 |
|  | neutral - positive | -0.013 | 0.012 | 0.583 |
|  | neutral - negative | -0.013 | 0.006 | 0.812 |
| DU | negative - positive | -0.010 | 0.011 | 0.540 |
|  | neutral - positive | -0.014 | 0.010 | 0.668 |
|  | neutral - negative | -0.013 | 0.011 | 0.641 |
| UD | negative - positive | -0.022 | 0.009 | 0.820 |
|  | neutral - positive | -0.019 | 0.021 | 0.500 |
|  | neutral - negative | -0.021 | 0.010 | 0.786 |

The equivalence tests results for RT of completing the MCE task were shown in Table S4. The TOST interval was [100, 100], the 95% confidence interval, and the total *p* value under different conditions were as follows.

Table S4 The 95% confidence interval on the difference between the means

|  | | Lower bound  (95%) | Upper bound  (95%) | The total *p*-value |
| --- | --- | --- | --- | --- |
| strategy | DU - UD | -360.826 | 235.568 | 0.859 |
| DU | negative - positive | -208.670 | 339.815 | 0.598 |
|  | neutral - positive | -18.498 | 622.045 | 0.893 |
|  | neutral - negative | -91.830 | 564.232 | 0.794 |
| UD | negative - positive | -282.125 | 293.125 | 0.742 |
|  | neutral - positive | -96.247 | 573.862 | 0.794 |
|  | neutral - negative | -100.021 | 566.637 | 0.786 |
